# Acceptability, Validity, and Engagement With a Mobile App for Frequent, Continuous Multiyear Assessment of Youth Health Behaviors (mNCANDA): Mixed Methods Study

**DOI:** 10.2196/24472

**Published:** 2021-02-10

**Authors:** Kevin M Cummins, Ty Brumback, Tammy Chung, Raeanne C Moore, Trevor Henthorn, Sonja Eberson, Alyssa Lopez, Tatev Sarkissyan, Kate B Nooner, Sandra A Brown, Susan F Tapert

**Affiliations:** 1 Department of Psychology University of California San Diego La Jolla, CA United States; 2 Department of Psychiatry School of Medicine University of California San Diego La Jolla, CA United States; 3 Department of Family Medicine and Public Health University of California San Diego La Jolla, CA United States; 4 Department of Psychology Northern Kentucky University Highland Heights, KY, CA United States; 5 Department of Psychiatry Institute for Health, Healthcare Policy and Aging Research New Brunswick, NJ United States; 6 Department of Music University of California San Diego La Jolla, CA, CA United States; 7 Department of Data Science and Operations University of Southern California Los Angeles, CA United States; 8 Department of Psychology California State University Los Angeles Los Angeles, CA United States; 9 Department of Psychology University of North Carolina Wilmington Wilmington, NC, CA United States

**Keywords:** mobile applications, young adults, smartphone, health behavior, underage drinking, alcohol drinking, self-report, illicit drugs, mobile phone

## Abstract

**Background:**

Longitudinal studies of many health behaviors often rely on infrequent self-report assessments. The measurement of psychoactive substance use among youth is expected to improve with more frequent mobile assessments, which can reduce recall bias. Researchers have used mobile devices for longitudinal research, but studies that last years and assess youth continuously at a fine-grained, temporal level (eg, weekly) are rare. A tailored mobile app (mNCANDA [mobile National Consortium on Alcohol and Neurodevelopment in Adolescence]) and a brief assessment protocol were designed specifically to provide a feasible platform to elicit responses to health behavior assessments in longitudinal studies, including NCANDA (National Consortium on Alcohol and Neurodevelopment in Adolescence).

**Objective:**

This study aimed to determine whether an acceptable mobile app system could provide repeatable and valid assessment of youth’s health behaviors in different developmental stages over extended follow-up.

**Methods:**

Participants were recruited (n=534; aged 17-28 years) from a larger longitudinal study of neurodevelopment. Participants used mNCANDA to register reports of their behaviors for up to 18 months. Response rates as a function of time measured using mNCANDA and participant age were modeled using generalized estimating equations to evaluate response rate stability and age effects. Substance use reports captured using mNCANDA were compared with responses from standardized interviews to assess concurrent validity. Reactivity was assessed by evaluating patterns of change in substance use after participants initiated weekly reports via mNCANDA. Quantitative feedback about the app was obtained from the participants. Qualitative interviews were conducted with a subset of participants who used the app for at least one month to obtain feedback on user experience, user-derived explanations of some quantitative results, and suggestions for system improvements.

**Results:**

The mNCANDA protocol adherence was high (mean response rate 82%, SD 27%) and stable over time across all age groups. The median time to complete each assessment was 51 s (mean response time 1.14, SD 1.03 min). Comparisons between mNCANDA and interview self-reports on recent (previous 30 days) alcohol and cannabis use days demonstrate close agreement (eg, within 1 day of reported use) for most observations. Models used to identify reactivity failed to detect changes in substance use patterns subsequent to enrolling in mNCANDA app assessments (*P>*.39). Most participants (64/76, 84%) across the age range reported finding the mNCANDA system acceptable. Participants provided recommendations for improving the system (eg, tailoring signaling times).

**Conclusions:**

mNCANDA provides a feasible, multi-year, continuous, fine-grained (eg, weekly) assessment of health behaviors designed to minimize respondent burden and provides acceptable regimes for long-term self-reporting of health behaviors. Fine-grained characterization of variability in behaviors over relatively long periods (eg, up to 18 months) may, through the use of mNCANDA, improve our understanding of the relationship between substance use exposure and neurocognitive development.

## Introduction

### Background

Obtaining behavioral reports on a mobile technology platform from youth has been widely applied in various substance use research contexts [1-5]. These reports are commonly tied to substance use interventions [6,7]. Mobile apps have also been used in community-based research on substance use [2,8,9]. The high penetration of mobile technology among teens enables this approach to be feasible [10]; however, frequent assessment of substance use in multi-year studies across a broad age group of youths has rarely been employed or evaluated.

More frequent assessment, over shorter reference periods in community-based research with youth, may provide 2 benefits: (1) allow for fine-grain characterization of substance use patterns, which can be complex among community youth [11], and (2) provide additional follow-up at a low cost and with minimal participant and staff burden. These advantages have important implications for large-scale longitudinal studies [12,13], such as NCANDA (National Consortium on Alcohol and Neurodevelopment in Adolescence) [12]. Youth in the NCANDA study have been assessed annually for ≥6 years on neurocognitive functioning, brain structure and function, psychosocial functioning, and various health behaviors and exposures, including substance use [12]. Substance use measures in NCANDA focus on aggregated substance use during the previous month and year during semiannual interviews. NCANDA’s investigation of the causal interplay between substance use and neurodevelopment in human youth would benefit from a more fine-grained assessment of substance use patterns during a critical period of on-going brain maturation.

Self-reporting of substance use has provided acceptable reliability and validity in certain contexts [14]. Factors that increase the validity of self-reported substance use include assurance of anonymity and confidentiality [14], computerized self-report (vs interview) [15], and the absence of sanctions tied to self-report of substance use [16,17].

Sources of error that affect the validity of self-reported substance use include the cognitive limits of recall. Specifically, respondents may have difficulty remembering the date in which events occur as the length of reference periods increase [18,19]. In addition, substance use associated with memorable events may be more easily recollected, contributing to bias [18]. Importantly, when the reference period captures more than 7 events, respondents tend to shift away from enumeration and move toward reporting substance use schema, which degrades validity [18,19]. Periods of abstinence or rare events of extreme use may be underweighted in participant reports. Perceived modal use patterns (ie, schema) rather than responses based on each of the particular substance use events during the reporting period will be more likely to become the basis of behavioral reports [14]. This previous work suggests that the assessment of substance use via self-reporting over an extended duration would benefit from the use of frequent reports based on relatively short reference periods. The design of the mNCANDA (mobile National Consortium on Alcohol and Neurodevelopment in Adolescence) app’s schedule for survey administration addresses the need to frequently collect self-report of substance use, maximizing assessment yield, given the constraints of cognitive recall and its effect on the validity of self-report.

**Figure 1:**
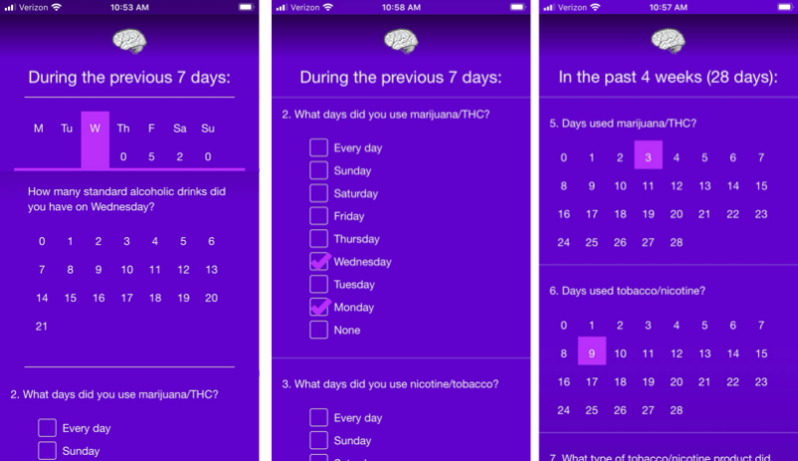
Examples of the mobile National Consortium on Alcohol and Neurodevelopment in Adolescence user interface. In the first panel, users enter the number of drinks they had consumed in each of the previous 7 days, starting with Sunday. This assessment would be issued on a Monday. Upon selecting a number of drinks, the value is entered into the day-by-day list and then automatically advances to the next day of the week until consumption on each day is reported. Users can revise entries by selecting the day. In the second panel, the user selects the day of the week that a substance was used. The last panel is the interface for reporting the frequency of use for 2 substances at a time in the previous 4 weeks, which is part of the core assessments. mNCANDA: mobile National Consortium on Alcohol and Neurodevelopment in Adolescence; NCANDA: National Consortium on Alcohol and Neurodevelopment in Adolescence.

To optimize long-term acceptability and respondent compliance to the mobile protocol for long-term, frequent assessment in NCANDA, a tailored app (mNCANDA) was developed. The app’s interface minimizes the number of actions for the participant to initiate and complete each assessment. For example, some items automatically advance to the next item upon response entry, and participants would be given *one-touch* response grids that allow a response to be selected again with a single touch rather than by typing several digits, scrolling, or dragging (Figure 1). Substance use assessment in mNCANDA is adaptive, meaning that fine-scale (day-to-day) substance use assessment does not begin until participants report more than 1 substance use event a month. In this case, alternative non–substance use–related items (eg, physical activity) are presented in lieu of substance use to equalize the assessment effort across participants. In addition, only commonly used substances (eg, alcohol, cannabis, and nicotine) were explicitly included in item prompts. Other substances were entered as a free response on their first report, but thereafter, those substances were explicitly included in item prompts and tailored to the participant’s vernacular. mNCANDA assessments were designed to take under 1 min each to increase compliance, and participants are given most of the day to respond. Together, these features may enhance the research value of mobile app–administered self-reports.

### Objectives

This study examined (1) test-retest reliability and concurrent validity of mNCANDA substance use behaviors, focusing on the most prevalent substances (alcohol and cannabis), (2) response rates over extended mobile follow-up assessment, (3) reactivity to the frequent assessment of substance use behaviors over an extended period of self-monitoring, and (4) usability and acceptability of the mNCANDA app, including user suggestions for fine-tuning it. Since the NCANDA project participants span several important development transitions (eg, end of high school and legal drinking age), an overarching study aim is to evaluate the extent to which the results varied as a function of age, which has implications for age-specific refinements of app design and content.

## Methods

### Recruitment and Eligibility

All participants (N=830) enrolled in the NCANDA project [12] were eligible for enrollment in the mobile app component of the study (mNCANDA), and 534 of these participants enrolled in mNCANDA. Initiation of recruitment to mNCANDA was staggered across the 5 NCANDA study sites (UC San Diego, SRI International, Oregon Health Sciences University, Duke University, and University of Pittsburgh) between April 2019 and June 2020. Recruitment was not complete when this analysis was conducted. Recruitment was limited to regularly scheduled semiannual NCANDA visits. As 3 sites initiated recruitment late and 1 was understaffed during much of the planned recruitment period, not all eligible participants had been invited to participate by the close of the initial evaluation period. The participants’ age range was 17-28 years. Tablets were available for participants who did not possess a mobile device. The mobile app protocol was approved by each research project site’s institutional review board, and all participants provided informed consent or assent with parental permission. Detailed demographic and substance use characterizations of the NCANDA participants have been previously reported [12].

### mNCANDA App

The mNCANDA app (Android and iOS) was engineered to provide participants with a low burden experience to obtain weekly behavioral and substance use exposure reports; the app was installed on participants’ personal smartphones. Examples of the interface are shown in Figure 1. No response data were stored locally on the phone or were accessible through the app interface. The software was impacted by a bug that limited participants’ ability to complete surveys during 2 assessment windows, which were dropped from the analyses (ie, treated as missing data). The app and protocol were developed in the context of iterative piloting that included both quantitative and qualitative feedback from youth pilot testers (see Mobile App–Related Attitudes and Perceptions: Usability, Acceptability, and Obtrusiveness below).

### mNCANDA Standard Assessment Protocol

mNCANDA issued weekly assessments, in addition to a core substance use assessment every 4 weeks. Weekly assessments were issued on Monday mornings, and the core substance use assessments were given on Sunday mornings. When the participant opened the mNCANDA app, the assessment was automatically loaded and presented questions. Assessment initiation (ie, when questions were seen by the participant) and survey completion times were recorded.

Participants would have approximately 14 h to complete the assessments after the first (morning) signal. If participants did not complete the survey after the first signal, the reminder signals would follow at 3 intervals after the assessment was first issued: 15 min, 5 h, and 6 h. The initial signal and the second reminder came as operating system notifications (banners), whereas the first reminder was delivered as a text message, and the final reminder was an email. Different signaling modes were used to protect against one of the modes failing because of an event such as a change in a handset causing loss of notifications or phone number change resulting in the loss of text signals. Each of these signals allowed the participant to know how much time remained before the assessment window closed.

The mNCANDA weekly assessments covered the following domains: substance use, access to substances, daily activity, stress, socialization, physical activity, use of complementary and alternative medicines, and quality of life. Groups of items were randomly administered each week to help maintain participant interest and engagement with the app, allowing for various developmentally relevant domains to be assessed without a substantial burden on the participant. Weekly substance use questions were granular down to the scale of individual days and were issued only to participants with a history of substance use, as reported in the mNCANDA core assessment. The items were adopted from the standard NCANDA battery and developed or adapted specifically for use on the mNCANDA platform. Items are outlined in Multimedia Appendix 1. Most items were adopted or adapted from several sources to conform to the objectives of the current protocol [20-25]. Content within the mNCANDA weekly assessments varied from week to week; groups of items related to the same domains were randomly issued on a particular week. Each of these question groups was composed of a small set of items (Multimedia Appendix 1). Most assessment events included the administration of 1 question group; 2 groups were issued simultaneously at a target rate of 33% of the assessment events.

The mNCANDA core substance use assessment evaluated substance use frequency (alcohol, nicotine, cannabis, and other drugs) and alcohol intensity during the previous 28 days. Participants entered other drugs as free-response entries. The frequency of substance use was measured in terms of the number of days with use during the reference period. The specific prompts for the items are included in Multimedia Appendix 1.

Participants were given incentives for the submission of mNCANDA assessment responses. The project used 2 incentive systems: threshold and ramped, which were randomly assigned. Incentives in the threshold schedule were US $2 per submission with US $5 to achieve a 90% response rate and an additional US $10 bonus for a 100% response rate during a set 8-week period. By comparison, the ramped schedule increased the incentive (approximately US $0.4) for each consecutive completed assessment in an uninterrupted series of submissions until a ceiling at US $4 was reached. In the ramped schedule, the sequence to determine compensation is restarted upon the participant missing an assessment. Under both systems, participants were presented with an interactive animation of coins showing the amount of their incentive being added to their collection immediately after each submission. The value of future submissions was presented to the participant in conjunction with the animation. The maximum total compensation in the ramped system is pegged to match the maximum under the threshold bonus system.

### Reliability and Validity Protocols

A standard core substance use assessment was issued via the app twice on the same day (Sunday) to evaluate the mNCANDA substance use assessment’s test-retest reliability. Participants’ response windows for the test-retest assessments were 4-h windows with one assessment given in the morning and the other during the afternoon with a minimum 1-h gap between assessments.

**Figure 2:**
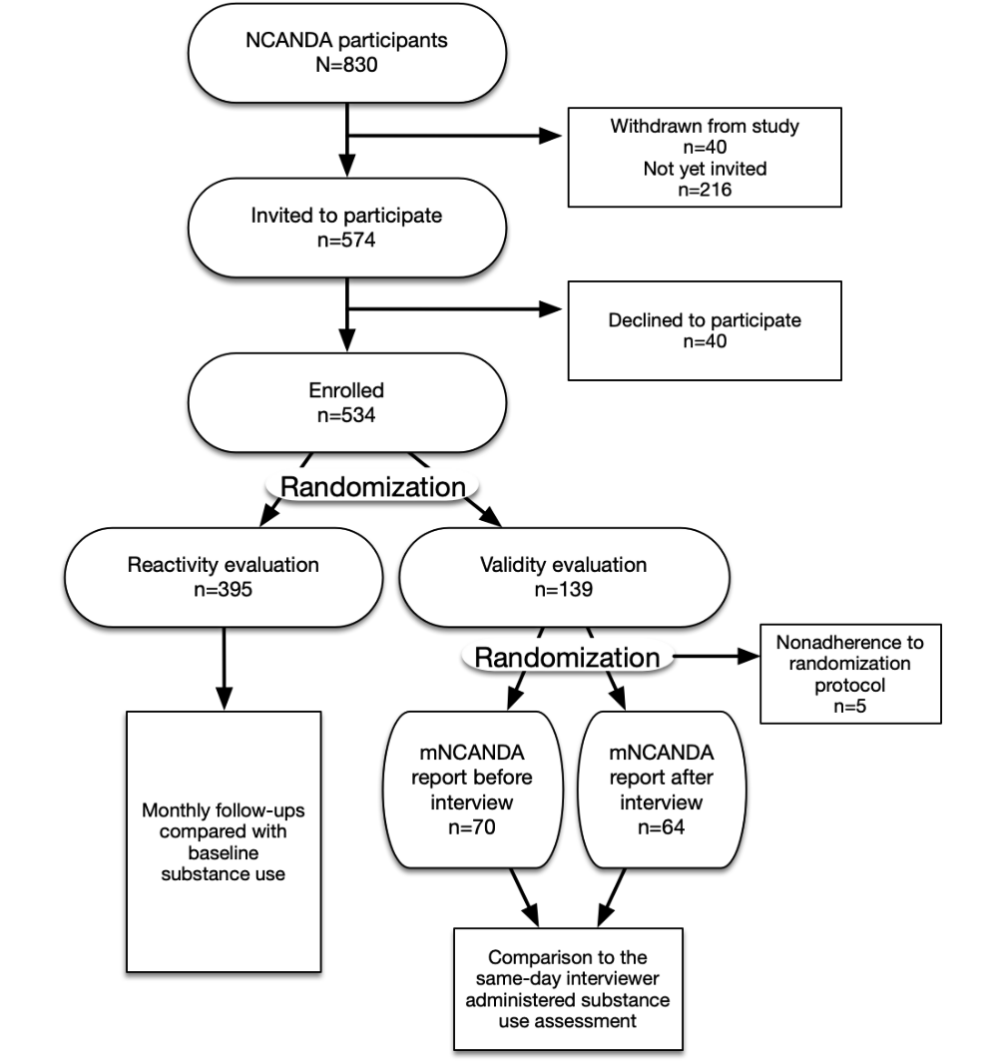
Participant recruitment, study allocation, and treatment allocation flow diagram. All National Consortium on Alcohol and Neurodevelopment in Adolescence (NCANDA) participants were eligible for participation. Recruitment invitations were issued during standard NCANDA interviews. Recruitment, consenting, and initiation of the mobile National Consortium on Alcohol and Neurodevelopment in Adolescence (mNCANDA) app takes approximately 20 min. The mNCANDA evaluation period ended before all participants were invited. mNCANDA: mobile National Consortium on Alcohol and Neurodevelopment in Adolescence; NCANDA: National Consortium on Alcohol and Neurodevelopment in Adolescence.

To examine concurrent validity, the mNCANDA self-administered self-reports were compared with self-reports obtained from the Customary Drinking and Drug Record (CDDR) [25], an established interview with known psychometric properties, which is used for the assessment of substance use. Approximately one-quarter (139/534, 26%) of participants were randomly selected to be included in the validity component of the study (Figure 2). These participants were then randomly assigned to receive their first mobile app assessment before or after an interviewer-administered CDDR was completed. This interview was scheduled as a standard component of the semiannual assessments issued in the parent study, which alternated between over-the-phone and in-person settings. Participants who were assigned to a particular assessment mode sequence reported substance use using a 30-day reference frame on their first assessment to match the CDDR’s reference period. The remainder of the participants used the standard app reference period of 28 days, which allowed for comparison with baseline use in the reactivity analysis.

### Reactivity

For participants randomized to the reactivity protocol (Figure 2), reactivity was assessed by evaluating changes in participants’ substance use in their initial report. The initial report assessed substance use in the month before enrolling in the mobile app protocol. Thereafter, participants’ substance use was assessed every 4 weeks. Reactivity was also addressed with 2 questions presented to the participants as part of the one-time feedback survey (see Mobile App–Related Attitudes and Perceptions: Usability, Acceptability, and Obtrusiveness below). Participants were asked how much they agreed with the statement, “Completing the surveys changes how I think about using alcohol,” and a separate statement that referred to “drugs.”

### Mobile App–Related Attitudes and Perceptions: Usability, Acceptability, and Obtrusiveness

Experiences and attitudes related to the app were evaluated using mixed methods. The specific mixed methods design structure employed was a concurrent nested design with embedded qualitative components within a quantitative investigation. A 20-item web-based, self-administered questionnaire was issued to all participants as part of the feedback survey. The feedback survey was issued in 1 bolus. At the time of survey completion, participation in mNCANDA ranged from 2 to 46 weeks (mean 25.4, SD 12.3). For each participant, survey items were randomly selected from a pool of 36 items related to usability, acceptability, obtrusiveness, item clarity, notification systems, incentives, and motivation. Many of the items were modified from or aligned with content found in several previous studies [26-28]. With the paucity of widely adopted instruments holding adequate content validity for each application, it remains common for usability studies to create usability instruments tailored to the specific project [29]. The items in this study assessed agreement with the item prompt on a 5-point Likert scale. In addition, open-ended items were included to provide capacity for interpretation, elaboration, identification of specific issues, and participant-suggested solutions. These open-ended items asked participants to describe why they did not respond to an assessment, the app’s features that they appreciated, and how the app and protocol could be improved. Qualitative components of usability investigations are critical for the identification of specific usability strengths and weaknesses [30].

A semistructured qualitative interview was also conducted with 9 participants, exclusively from the San Diego site. Participants were selected through purposeful sampling [31] so that the maximal number of intersections among the 4 participant characteristics was obtained. These characteristics were sex (4 female vs 5 male), age (5 under 21 vs 4 over 21), substance use (4 naïve vs 5 experienced), and mNCANDA response rates (6 high responders vs 3 low responders). Descriptions of participants’ experiences using the mNCANDA app were elicited. These semistructured interviews were organized around an interview guide (Multimedia Appendix 2). Interviews were conducted by a research assistant who had previously developed a rapport with the participants while conducting prior interviews. Interview length ranged from approximately 10 to 20 min. Responses were audio recorded, transcribed, coded, and evaluated for the emergence of themes. Coding was conducted by a senior graduate assistant with training in qualitative analysis. Identification of themes was developed in consultations between the first author and the coder, from a grounded theory perspective [31]. Using a complementary mixed methods approach [32,33], the qualitative data were used to give context, elaborate beyond the quantitative response patterns, and screen for critical aspects of the user experience that were absent from the quantitative assessment.

### Statistical Analysis

Test-retest reliability was examined using intraclass correlation. Concurrent validity, the extent of agreement between mNCANDA self-reports and the 30-day CDDR of alcohol and cannabis use frequency, was assessed using Bland-Altman plots [34,35] and ordinary least squares regressions with difference scores modeled by the mean of the 2 measures, an indicator for the measurement order, participant age class, and their interactions. Age was categorized as below 18, 18-20, and above 20 years in the statistical models, which is consistent with previous NCANDA age categorization [12] and captures common developmental thresholds. This categorization was a preplanned analytic approach. The exploration of age as a continuous variable was also conducted as a secondary exploratory step, in part motivated by the small numbers of participants in the below-18 years age category by the time the app was deployed and human research protection program review and approval could be obtained.

To examine long-term response rates (ie, engagement) with the mNCANDA app and its correlates (eg, differences in response rates over time by age), generalized estimating equations (GEEs) for binomial outcomes with an autoregressive function for the errors were used. GEEs assume that data are missing completely at random, which is appropriate for these data with monotone missing patterns structured by design. The dependent variable was the completion of the mNCANDA assessment (yes/no). The GEE model included the sequential assessment number (nested within participants), age, and their interaction. GEE models were fit using iteratively reweighted least squares.

Mixed effects Poisson models were used to evaluate assessment reactivity or changes in substance use after initiating the mNCANDA protocol. These models assume that missing values are missing at random. The dependent variable in these models was substance use frequency (alcohol and cannabis). Frequency of use was modeled as a function of the calendar month (to account for seasonal patterns in substance use across the year) and the sequential assessment number (ie, time). An index for participants was included as a random effect. A second set of models extended this base model. This included an indicator variable signifying whether participants self-reported on the feedback survey that they perceived the mNCANDA protocol influenced their substance use. An interaction term between the assessment sequence number and self-reported reactivity items was also included as a step in the hierarchical model building. As participants initiated the protocol on a rolling basis, systematically determined by participants’ NCANDA interview schedules, statistical control of temporal patterns in substance use across the year was incorporated into these models by including calendar month as an independent variable. Models were estimated via maximum likelihood. The sets of model parameters added in each step were evaluated using likelihood ratio tests. Analysis of the quantitative survey items consisted of contingency tables for the relationship between age class and response categories using Fisher exact tests.

## Results

### mNCANDA Sample

At the end of the evaluation period, 72.7% (574/790) of the active NCANDA participants had been approached to participate in the mNCANDA protocol, and most (534/574, 93%) consented and were enrolled. All participants possessed a smartphone. About half (225/534, 42.1%) of the mNCANDA sample was female. At the initiation of the mNCANDA study, participants had a mean age of 21.6 years (SD 2.5), representing the ages of 17 (21/534, 3.9%), 18-20 (187/534, 35%), and 21-28 (326/534, 61%) years. The self-reported race of the mNCANDA sample was 73.6% (393/534) European American, 9.7% (52/534) African American, 7.9% (42/534) Asian, 0.6% (3/534) American Indian, 0.6% (3/534) Pacific Islander, and 7.7% (41/534) mixed or other race or ethnicity; 15.9% (85/534) identified as Hispanic. Compared with the overall pool of NCANDA participants, the enrolled mNCANDA participants were similar to the larger pool based on sex (51% female; Fisher exact test, *P*=.16), age (21.6 years; t_827_ value=0.96; *P*=.36), and race (72% European American, 12% African American, and 8% Asian; Fisher exact test, *P*=.19). Most (422/534, 79%) participants reported some substance use via the mobile app throughout the study. Many (368/534, 68.9%) participants also reported using alcohol in the previous month at the baseline mobile assessment. Fewer participants (198/534, 37.1%) reported using cannabis at the baseline assessment.

### Adherence and Time to Complete the mNCANDA Assessment

The median time it took participants to open an mNCANDA assessment, generate responses, and submit survey responses was less than 1 min for most weekly and core assessments (N_assessments_=6113; median 51 s; IQR 0.85 min). The mean response time was 1.14 min (SD 1.03 min).

**Figure 3:**
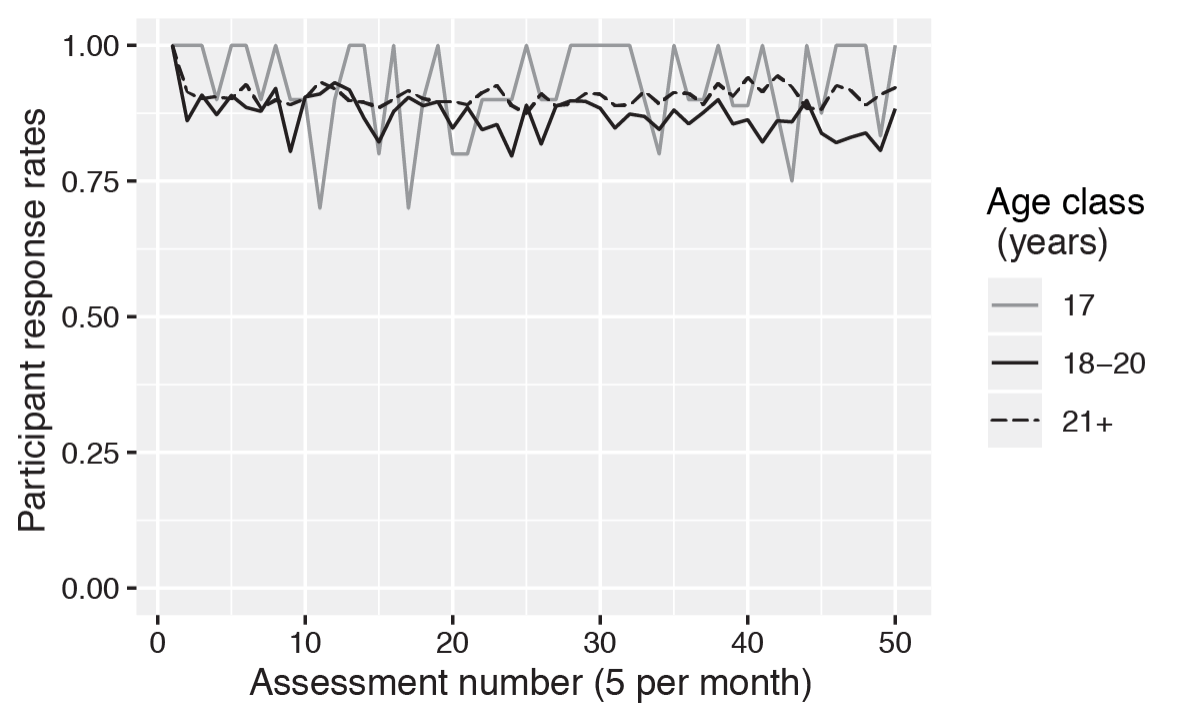
Mean response rates over time by age class. Assessment number is the sequential index assigned to each participants' series of scheduled assessments.

Participants were assessed continually (ie, at least weekly) with mNCANDA for up to 18.6 months. The mean assessment period was 8.2 months (SD 4.3). The mean response rate was 82% (SD 27%), with 61% (326/534) responding to at least 90% of the study’s scheduled assessments. Evidence of an association between age and response rates was absent (*P*>.35). Response rate fluctuations among all age classes predominantly remained above 80% (Figure 3). Evaluation of the temporal patterns of response rates in a GEE model failed to identify temporal trends among any of the age classes (*P*>.20). The strongest age parameter in the GEE models was for the interaction between the assessment number and the 18-to-20-year-old age class (older than 20 years was the reference group; *b*=−.004; SE 0.003).

During the semistructured qualitative interviews, participants indicated that some of the common reasons for missing surveys involved employment, socializing with family and friends, when the assessment notifications were sent, and getting distracted and forgetting to return to the assessment. The self-administered questionnaire also included several noteworthy, isolated responses. These included parental confiscation of the participants’ phone, stolen phone, traveling where internet access was limited, and choosing not to respond when the participant was not confident in accurately recalling events. For example, a respondent wrote, “If I don't remember answers to questions, I usually don't answer. Asking me to recall the past month of alcohol use is difficult” (Survey Participant 1). Several respondents also indicated that, on occasion, mobile app “glitches*”* prevented them from completing an assessment.

A robust theme in the qualitative open-ended responses related to engagement with mNCANDA was the brevity of the assessments. “The questions are easy to understand, there are an appropriate amount of questions, so it doesn’t take up too much time,” (Survey Participant 2) was characteristic of the explanation that many participants used as a reason for liking the system. “Straightforward,” (Survey Participant 3) “simple,” (Survey Participant 4) and “it doesn’t take much time” (Survey Participant 5) also tie in with a theme identified in the interviews. Interviewees described brevity as one of the reasons they were agreeable to the proposition of continued long-term participation in the mobile study.

### Reliability

Test-retest reliability was found to be high for substance use reports provided via the mobile app. A total of 260 participants (260/338, 76.9% of the active participants at the time) responded to the pair of test-retest assessments. Responses to the 30-day use items for alcohol and cannabis exactly matched for 81.9% (213/260) and 88.1% (229/260) of participants, respectively. The overall correspondence resulted in intraclass correlations of 0.969 (95% CI 0.961-0.977) and 0.987 (95% CI 0.983-0.989) for alcohol and cannabis, respectively.

### Concurrent Validity

**Figure 4:**
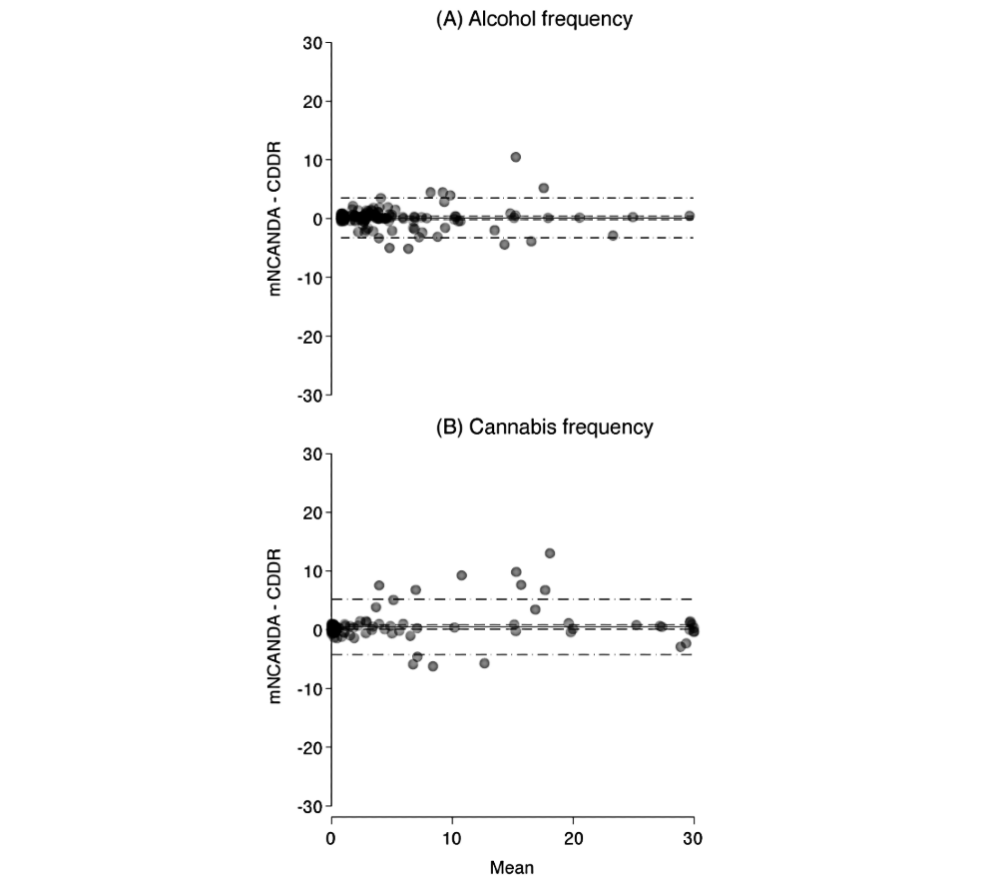
Bland-Altman plot comparing (A) alcohol and (B) cannabis use frequency as reported via mobile National Consortium on Alcohol and Neurodevelopment in Adolescence and in the Customary Drinking and Drug Record. Dashed lines are 95% CIs, and the dash-dot lines are the 95% limits of agreement. Markers are jittered (2%). CDDR: Customary Drinking and Drug Record; mNCANDA: mobile National Consortium on Alcohol and Neurodevelopment in Adolescence.

Comparisons between the mobile app and interview self-report on the recent (previous 30 days) alcohol and cannabis use days showed close agreement for most observations (Figure 4). The mean number of reported alcohol use days was higher by 0.13 (SD 1.7) days on the mobile app relative to the interview report (95% CI −0.17 to 0.42). No associations were detected in the regression model for the difference in the reports related to participants’ age, the mean of the 2 reports, and whether the mobile app report was issued before the interview (R^2^=2.9%; *F*_10,123_=0.33; *P*=.98). The mean number of cannabis use days was 0.48 (SD 2.4) times higher on the mobile app than that reported in the interview (95% CI 0.07-0.88). As with alcohol, no patterns were detected in the regression model predicting differences in cannabis use reports from the participants’ age, the mean report, and whether the mobile app report was issued before the interview (R^2^=3.7%; *F*_10,123_=0.48; *P*=.90). However, the limits of agreement were wider for cannabis than alcohol, with the largest discrepancies favoring greater use days on the mobile app (Figure 4). For both substances, most (92%) responses agreed without deviation at the minimum (0 days) and maximum (30 days). Where there was a deviation at these extremes (for either report), the most common (56%) magnitude of difference in the reports was 1 day of use.

### Reactivity

**Figure 5:**
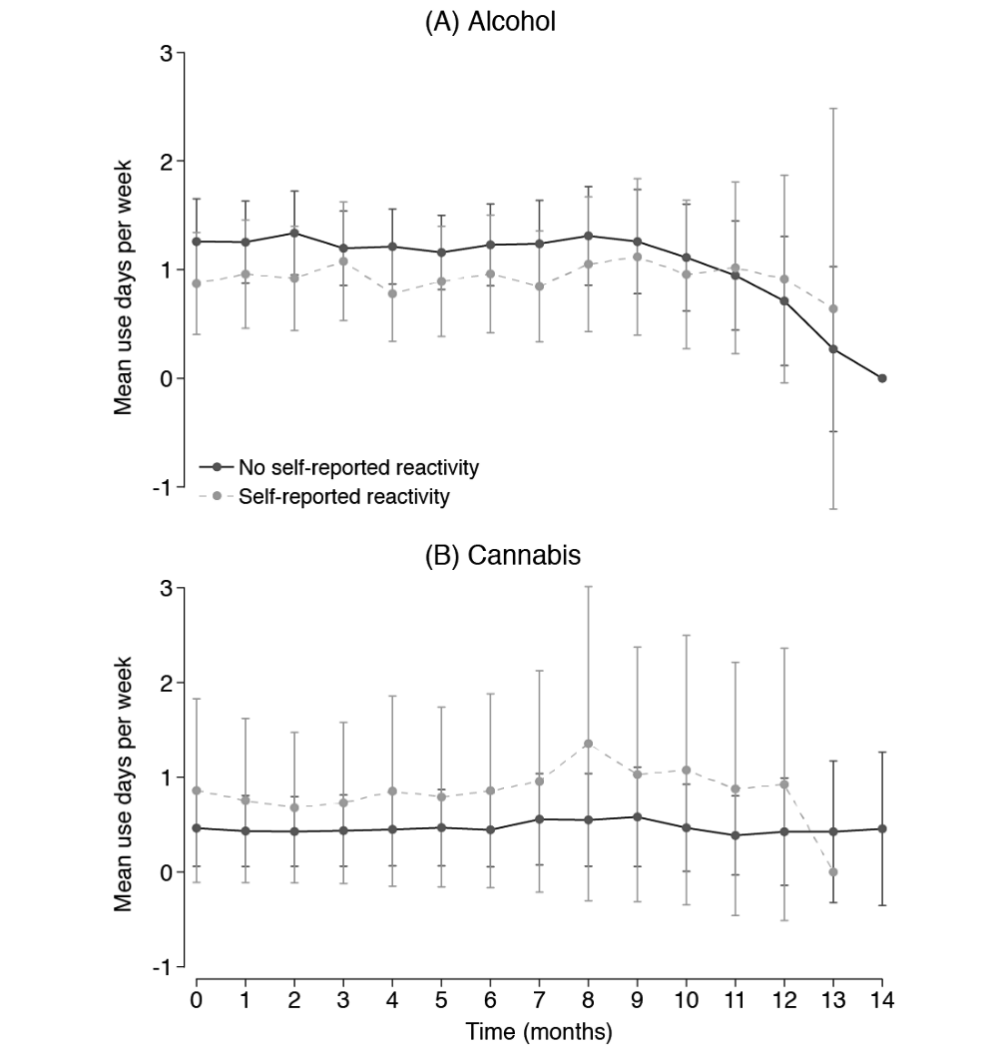
Days of substance use during 4-week intervals for alcohol and cannabis as a function time since the baseline and self-reported cognitive reactivity to the mobile app after adjustment for the calendar month. Error bars are 95% CIs around marginal means extracted from the mixed effects models. Time 0 assesses the month before the initiation of mobile National Consortium on Alcohol and Neurodevelopment in Adolescence assessments. mNCANDA: mobile National Consortium on Alcohol and Neurodevelopment in Adolescence.

Patterns of change in alcohol or cannabis use were not evident in the assessment of reactive changes in the frequency of substance use (Figure 5). In the reactivity models, there was no resolvable evidence of change over time; time parameters from models for both substances were not statistically significant (likelihood ratio *X*^2^_14_<15.0; n=395; *P*>.39). Of the 395 participants randomized to the reactivity evaluation, 165 were also randomized to receive items in the feedback survey asking if the repeated assessments changed the way they thought about their substance use. Among those 165, 38 (23%) reported that the repeated assessments changed how they thought about their substance use (Figure 5). When stratifying participants by self-reported reactivity, differences in trajectories were not evident (Figure 5); the interaction between time and self-reported reactivity was not statistically significant (likelihood ratio *X*^2^_13_<6.2; *P*>.93). Furthermore, age and its interaction with time did not show an association with patterns of alcohol or cannabis use (likelihood ratio *X*^2^_26_<4.1; *P*>.98).

A theme that emerged in the qualitative interviews was that the participants’ perception of their substance use behaviors did not change as a result of using the app. Some participants reported feeling more aware of their behaviors as a result of the app:

The questions about how much time I watch [television] or how I spend my leisure time, I tend to notice more of my habits. It hasn’t prompted any change, but I am more cognizant but doesn’t change anything.

Interview Participant 2

It made me a little more alert because when I get to the survey, I don’t remember if I did this or that. It just made me remember what I am doing more, like how—how much I am drinking or using and just makes me more observant and self-aware, I guess, of what I have been using.

Interview Participant 9

Although respondents stated they were more aware of their behaviors, this statement was followed by a denial that changes were made in their substance use patterns. This theme was not universally supported; an interviewed participant stated that they did reduce how much they drank because of the assessments (Interview Participant 5).

### Mobile App–Related Attitudes, Perceptions, and Beliefs

Pooled estimates across age groups are presented in this section. With 2 exceptions, support for differences in response patterns among age groups was not evident (*P*>.08).

#### Usability

**Figure 6:**
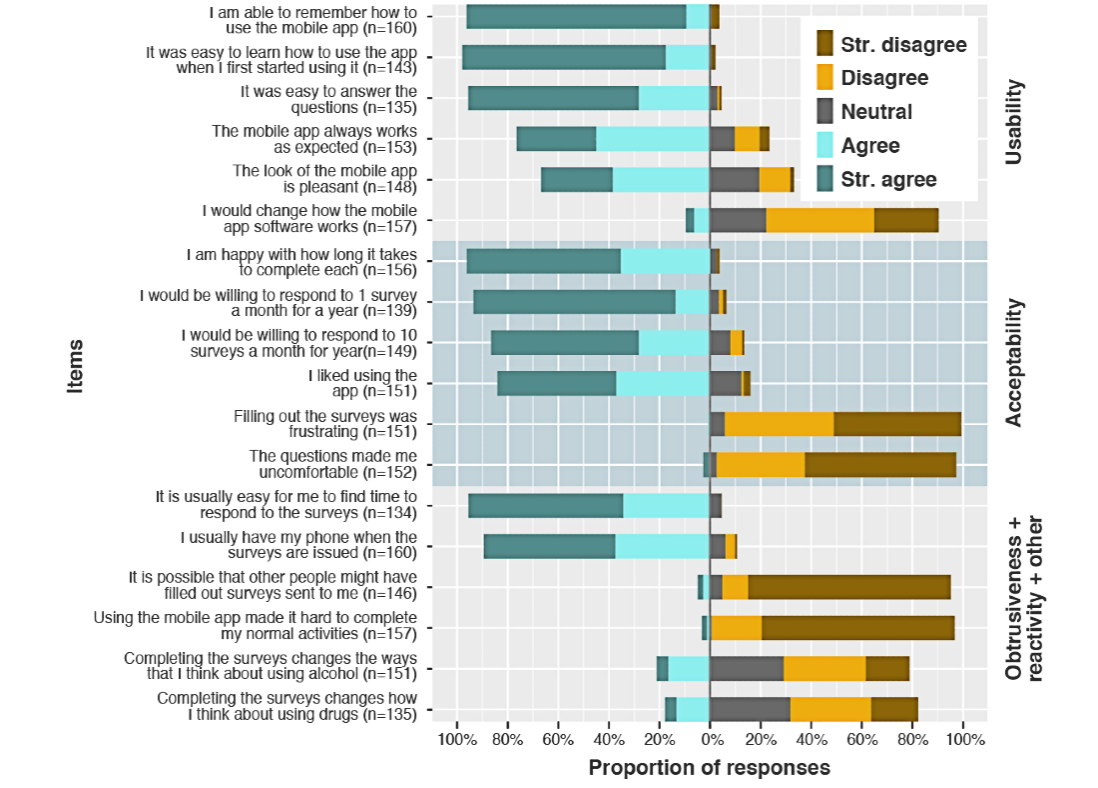
Participant responses to the closed-answer questions about perceptions and attitudes related to the mobile National Consortium on Alcohol and Neurodevelopment in Adolescence software and assessment protocol usability, reactivity, and acceptability. mNCANDA: mobile National Consortium on Alcohol and Neurodevelopment in Adolescence.

Most participants rated mNCANDA favorably on the dimensions of usability (Figure 6). More than 67% agreed to each of the positive items related to ease of use (Figure 6). By comparison, 9.6% (15/157) reported a desire for the app to be modified. However, only 3.2% (5/157) strongly agreed with this item. A minority (21/153, 13.7%) indicated that the app did not always work as expected.

The simplicity of the mNCANDA app was a theme in the qualitative data. However, multiple participants reported at least one time when the app crashed or glitched, and they were not able to submit or open the survey. A few participants had some suggestions regarding features to improve within the app, such as changing fonts and colors and adding a free-response item to allow for the elaboration of their responses. One of the interviewees stated, “I wish sometimes there was a button where I could explain maybe, like the circumstances of what was going on during that particular week” (Interview Participant 4). Interviewees expressed the recognition that some events may not be easy to categorize because they were unsure how the project administrators wanted unusual situations to be categorized.

An item related to usability evidenced differential response patterns across age classes assessed, “It was easy to answer the questions” (Fisher exact test, *P*=.03). Very few respondents disagreed with this statement at any age. The difference driving this finding was a greater proportion of 18-to-20-year-olds strongly agreeing (26/32, 79%) rather than agreeing (4/32, 10%). In the other age classes, the ratio of strongly agreeing to agreeing was 2:1.

#### Acceptability

Most (64/76, 84%) of the participants agreed to the positive acceptability items, including liking the app and willingness to continue with the app for another year (Figure 6). The proportion of participants strongly agreeing to another year of assessments did drop from 80% to 58% when participants were asked about reporting on 1 assessment a month compared with 10 surveys a month. Almost all participants (96%) agreed that they were happy with the length of time that the assessments took to complete.

In addition to the brevity theme, ease of use also emerged as a strong theme in the qualitative interviews. Participants attributed the brevity and ease of answering questions as aspects that increased their willingness to respond to the weekly mobile app assessment on a long-term basis. When asked what it would take for participants to continue using the app, participants stated:

honestly, I think what you guys are doing right now is perfect... It has been super easy and... like it isn’t taking time out of my day.

Interview Participant 6

I do like... how it is set up as is honestly... it seems easy to scroll down and navigate... I think if it stays more or less as is with the notifications and weekly assessments, I think I would be participating moving forward.

Interview Participant 2

#### Obtrusiveness

Almost all (129/134, 96.3%) participants strongly agreed that they could easily find time to complete the assessments. Only 1.9% (3/157) reported that they strongly agreed that the assessment protocol interfered with their normal activities.

#### Scheduling of Assessments

There were differences by age in responses to the item on the desire to change the days of the week on which assessments were scheduled (Fisher exact test, *P*=.02). For all age classes, few (<8%) participants reported a desire to change the scheduled days; however, differences in the rate of neutral reporting were apparent. The main difference across age was dominated by the large proportion (16/32, 50%) of participants under 21 years, indicating a neutral position on this item, whereas those over 21 years were uncertain only 27% (24/89) of the time. Those aged above 21 years were more likely to report agreeableness with the assessment days than the younger participants (63/89, 71% vs 15/32, 47%).

#### Item Clarity

**Figure 7:**
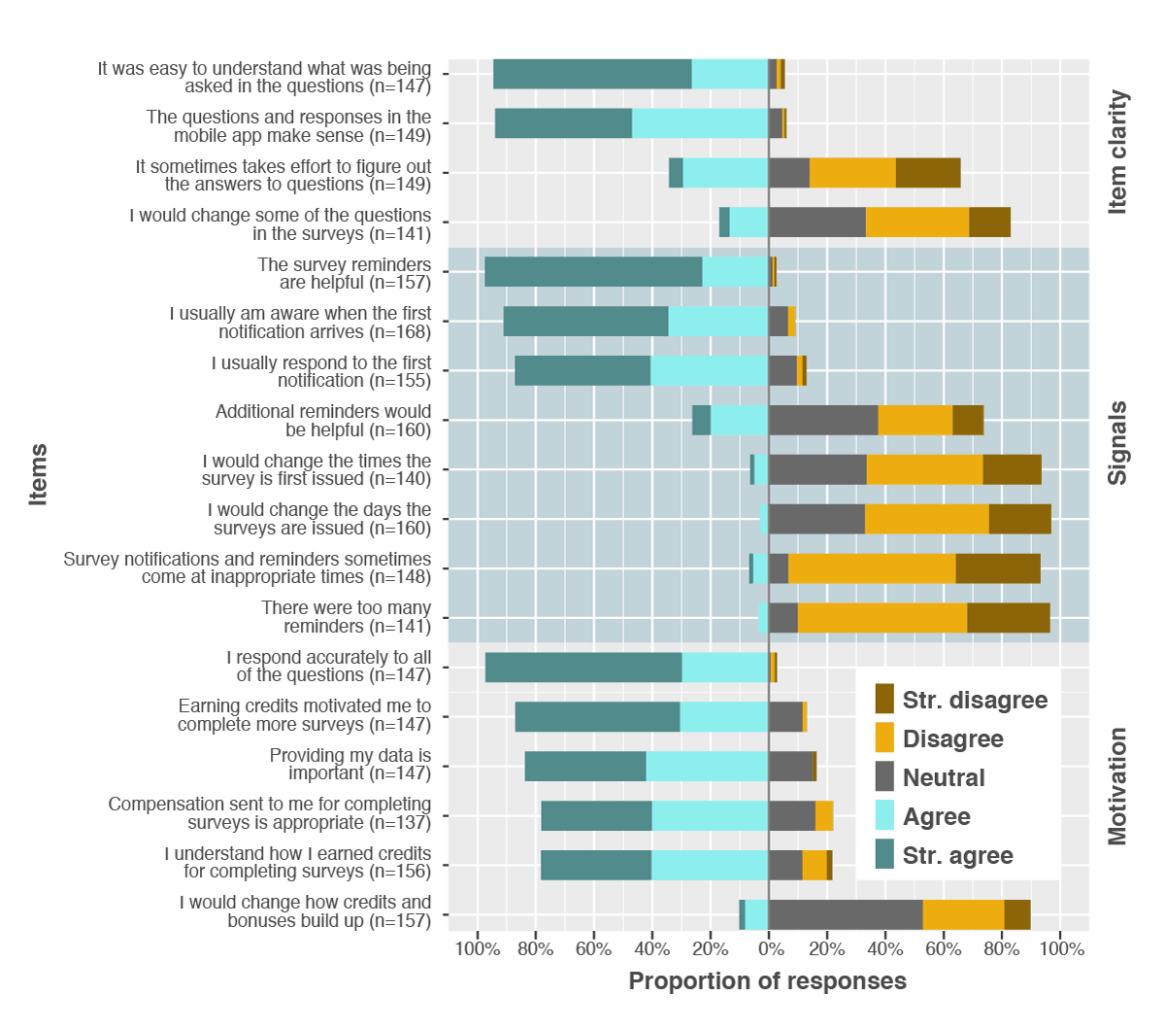
Participant responses to the closed-answer questions about perceptions and attitudes related to the mobile National Consortium on Alcohol and Neurodevelopment in Adolescence item clarity, signals, and motivation to participate. mNCANDA: mobile National Consortium on Alcohol and Neurodevelopment in Adolescence.

Most (70%) participants reported understanding the items as they were presented (Figure 7). Only a minority (17%) reported that they would change some of the questions.

Regarding item clarity, 2 themes emerged in the qualitative data: reference period and relevance. Several participants were unclear if behaviors that occurred on the same day an assessment was issued should be included in the reference period for the behaviors being assessed. The item prompts participants to report behaviors that occurred during the previous 7 days, for example. Another theme was relevance. Participants felt unsure about how to respond to items that were not relevant to their own lives. The examples provided were questions about work and school, which were not seen as relevant to participants not working or not in school, respectively. Certain assessment items were also identified as being difficult for the participants to understand, with a common focus on the items asking participants to identify their social networks. Participants reported that this might be construed in various ways.

#### Signals and Motivation

Most (152/157, 96.8%) participants strongly agreed that reminder signals were helpful (Figure 7). Very few (6/141, 4.3%) participants indicated that there were too many reminders. There were also patterns of responses across reminder-related items, indicating that most participants were not distressed by the reminder regime (Figure 7). The patterns of responses to items related to motivation indicated that participants responded accurately and attributed the incentive system to at least some of their motivation to complete the surveys (Figure 7).

The qualitative interviews revealed substantial diversity in preference for the timing, form, and nature of the reminder notifications. A participant stated, “I like that they are sent pretty early in the morning...” (Interview Participant 2). Another stated, “I would say that I like noon-ish” (Interview Participant 8). Some participants claimed, “...the text message is most effective...” (Interview Participant 5). Others stated that they liked the app notification “because from there you can automatically click it and go to the app” (Interview Participant 8). A theme that arose in most of the interviews was the desire for the app to issue a final reminder near the end of the reporting window when they had not already responded. For example, a participant suggested, “having second notifications... to remind you... probably like 30 minutes to the deadline” (Interview Participant 6). When interviewees were asked if the number of notifications was intrusive, none thought that they were.

### Suggested Improvements for the mNCANDA App

The qualitative data included several themes related to areas for improvement. A strong theme about the system not needing improvement dominated the self-administered open-ended responses. Some respondents suggested changes. One of them was to limit the reference period for behavioral items to the previous week by eliminating items referring to the previous month. Respondents explained that they had more confidence and found it easier to report the previous week’s behaviors. Several respondents asked that the response format be based on a calendar or have it formatted with the date so that individual events could be used to cue and record their activities. A theme of user tailoring arose, where users’ ability to adjust the timing of survey notifications was seen as a potential benefit to the project.

## Discussion

### Participant Engagement

Mobile app assessment of substance use has been found to be a highly feasible and valid method among various clinical and nonclinical adult populations [6,7,36-38]. This includes a diverse collection of apps used for alcohol interventions with youth [6,39]. However, these are often of shorter duration (weeks to months) and are aimed at a targeted population seeking assistance [6,7]. Nevertheless, the retention of participants at 80% or higher is inconsistently attained, and response rates exceeding 80% in studies extending beyond a month were not identified [7]. To date, there has been limited research on continuous long-term mobile assessment for substance use among community adolescents and young adults. This population is particularly susceptible to problematic substance use, including high rates of use and substance use–related socioemotional harm [40]. Furthermore, previous studies have used a burst-design methodology, assessing real-time substance use among adolescents and young adults for a short period, usually less than 1 month over longer follow-up [2,8,9,41-43]. Our findings build upon this body of previous work by demonstrating feasibility, acceptability, and validity of smartphone-based measurement of substance use among American community youth 5 times per month over an extended period (eg, up to 18 months).

Impressively, adherence to the assessment protocol throughout the study was, on average, 82%, with 61% (326/534) of the participants responding to at least 90% of the assessments. There was no evidence of bias because of nonresponse over time associated with age. Contrary to our hypotheses, mNCANDA response rates did not decline throughout the extended follow-up. These findings support the use of frequent (eg, weekly), continuous smartphone-based mobile assessment in longitudinal studies of substance use in this high-risk age group. The fine-scale temporal characterization of substance use behaviors can help elucidate differential short- and long-term patterns of use and contextual factors impacting use behaviors over time, with minimal burden to the participant. This is particularly valuable during this developmental period, where substance use is known to fluctuate [2,11]. Low burden measurement approaches, such as mNCANDA, could provide a feasible means to increase the number of longitudinal studies that collect temporally fine-scale behavior data. With the app, the quality of estimated substance exposures over the total course of youth development is expected to be improved; it is expected that respondents will be more likely to enumerate substance use events that are present as salient and retrievable memories, reducing recall biases.

Alcohol consumption assessed in community samples is differentially underreported, particularly by those with lower consumption levels [44,45]. Protocols that allow adjustments to longer reference period reports of drinking frequency based on very recent (yesterday) recall are capable of reducing discrepancies between objective measures (eg, biomarkers) and self-reports [45]. Here, the mNCANDA protocol includes overlapping moderate (last month) and short (each of the last 7 days) reference periods. These reference periods should both reduce bias compared with yearly assessments and provide a fine-scale characterization of youth substance use.

### Acceptability and Engagement

This study’s high rates of engagement in terms of total participation and response rates are attributable to several factors. First is the simplicity and brevity of the assessments. Only a small number of simple items were presented at a time to the respondent. Most participants found the overall mNCANDA assessment length (~1 min to complete) acceptable. In addition, the user interface was designed to push the user along with a minimum number of user actions. Questions were rotated randomly, which guarded against monotony. Participants were also given an entire day to report; 3 reminders were sent via multiple modes, which was deemed acceptable by most users. The mNCANDA app was designed to reinforce participation by tying survey completion to an animation that appeared upon survey submission (ie, tokens filling the screen) and by providing compensation every 8 weeks (up to US $35) to demonstrate the value of their participation in the project goals. Taking all the mNCANDA app features together, most participants reported they liked the app, which contributed to high completion rates. The high response rates in the mNCANDA study were similar among adolescents, emerging adults, and young adults and maintained through the 18-month follow-up.

The mNCANDA sample was ascertained from a pool of participants who had already participated in a longitudinal study for several years. Retained participants have already invested in the project and may not be representative of the general population. Although the NCANDA participant pool is representative of the catchment areas near each of the study sites on most major demographic dimensions, the participants’ parents’ education levels are high (53% with at least one parent with education beyond an undergraduate degree) [12]. All participants in the study possessed their own smartphone, which is approaching universal adoption for adolescents [10]. However, adolescents are also exposed to developmental stage-related barriers in phone use, such as temporary parental confiscation of their mobile phone, which can affect timely assessment completion. In addition, the prevalence of smartphones among adolescents is lower in households with lower incomes and educational attainment [10]. In previous studies, access to a mobile device and reliable internet connectivity has been a barrier for some participants [5,46]. In a text messaging–based study, access to unlimited texting and data plans was associated with greater responding among youth from a resource-limited community [3]. A way to address this gap could be by providing rapid cost recovery to participants, which might help make engagement in the research project more equitable. The use of a mobile app that requires low bandwidth, such as mNCANDA, can further alleviate this issue.

Most participants endorsed responses that indicated acceptability of the mNCANDA app and protocol, indicating that they would be willing to continue for another year. Very few participants reported being frustrated by the app, even though over 10% reported that the app did not always work as expected, and there were 2 bugs that impacted all app users. The most troubling bug blocked users from downloading an assessment even after having been signaled repeatedly that the assessment was due. In the qualitative assessment, some participants mentioned mobile app glitches as a concern but remained forgiving of the frustrations. When users register for mNCANDA, they are informed that it is an in-house app that is still under development and provided with a way to report bugs.

Attrition and low engagement are general challenges for health-related mobile apps [47]. Usability issues are thought to be gatekeeping barriers to engagement [48]. There is a danger of overengineering and neglecting user preferences and needs [47]. Adoption of formal iterative development cycles can reduce the risks of unexpected usability barriers and optimize acceptability [49,50]. An example of iterated development was reported by Huguet et al [36]. mNCANDA was similarly developed with several development cycles punctuated by quantitative and qualitative feedback. The mNCANDA process used 4 evaluation methods and 2 approaches outlined by Moumane et al [51] to iterate to a version that is highly usable and acceptable. Usability is not a core focus of many research reports about mobile apps related to mental health [52], and many do not include or report the qualitative findings that are critical to guiding the iterations. Qualitative responses have been important in guiding the development of other mobile apps, and reporting of the related qualitative results provides useful lessons for designing future apps [36,46].

### Validity and Reliability

The respondents also demonstrated that they were attentive to providing reliable and valid responses. First, very few participants indicated that another person might have completed any of the surveys (there is currently no authentication step as a trade-off for simplicity and speed). The test-retest concordance was extremely high for self-reporting. This demonstrates that the mNCANDA system (ie, the user interface, item construction, and protocols) itself is constructed to promote reliable response entry, elicit attentive responding, and consistency. An additional factor that could explain the response patterns is that the app is being used in the NCANDA project context, which has focused on supporting its participants’ interest in maintaining scientific contributions and developing trusted partnerships. The concurrent validity of the mNCANDA responses was also found to be favorable when gauged against the standardized CDDR interview administered as a semistructured substance use assessment [25]. Although there was some variability, respondents provided comparable substance use reports between the 2 measurement techniques, particularly at the extremes, where formulating a response may be easier. Where there were deviations between the reports, they tended to favor greater reporting of substance use using mNCANDA. Although self-administration of substance use assessments has been found to result in greater disclosure [53], this was not expected for the NCANDA project. Social desirability biases are thought to be ameliorated in NCANDA, as participants are specifically enrolled in a substance use study where they are repeatedly asked about recent and lifetime substance use by professional research staff who interview participants regarding the details of their substance use. Overall, repeat testing and concurrent validity for the mNCANDA app were favorable.

### Reactivity

Evidence of substantial reactivity to the mNCANDA app measurements was absent. Substance use trends were steady when compared with the period before mNCANDA protocol initiation. Few participants reported conscious changes in their substance use behaviors that they attributed to the repeated self-report measures. Of those who reported that they reflected more about their substance use, there were no detectable changes in substance use after starting to respond to mNCANDA assessments. In a randomized study, Buu et al [54] found reactivity to a long-term repeated assessment of recent substance use was limited to the first week of monitoring, where the frequency of alcohol use increased but quantity decreased. In contrast, self-monitoring is a mechanism leveraged by substance use interventions [55,56]. However, NCANDA is not based on a clinical sample. Substance use is primarily treated as an exposure in the primary aims of the NCANDA project, so reactivity is of a lower concern than it would be in other research contexts. However, the use of mNCANDA increased exposure to harmful levels of substance use, an evaluation of the ethics of continuing to use the app would be warranted. The findings have implications for other youth substance use researchers. Some caution should be exercised when considering the frequent long-term assessment of youth based on the current data because this study is sensitive only to gross changes in substance use frequency. Furthermore, other features of a person’s pattern of substance use may have been altered without being detected.

### Participant Feedback

The mNCANDA participants provided numerous suggestions for ways to improve the system. Many of the isolated suggestions are being considered for incorporation into the future evolution of the app. Those that formed emergent themes include the request for tailoring the notification-reminder timing for each user. Ensuring that notifications arrive at opportune times rather than during contexts where it would be obtrusive would be a valuable enhancement to long-term assessment. For example, some users indicated that assessment signals arrived when they had been on duty at work, which is most relevant for young adults. In addition, to diminish nonresponse because of oversight, the suggestion to add a final notification near the close of the response window has been incorporated into the system. Allowing individuals to tailor their notification schedule could allow optimal timing of reminders while limiting obtrusiveness; negative affective reactions can be ameliorated by providing the user with greater control of the reminder signals. In the study reported by Huguet et al [36], approximately 40% of participants personalized the times at which they received their signals.

The nonrelevance of items was another theme; 2 approaches were incorporated into the mNCANDA protocol to address participant concern about receiving items that are not relevant for them. The first is item tailoring. For instance, substance naïve respondents do not receive regular assessments of their day-to-day substance use as part of the weekly assessments. Most of the items subject to nonrelevance concerns will continue to be issued, but with more regular reminders, a valid response can be provided for all items by all responders. For instance, users who do not have a job can respond that they have worked 0 h in the previous week. Some of our respondents did not recognize a report of 0 h as being an appropriate response, which may be an undetected issue in other behavior studies on North American youth.

Additions to the protocol would need to be balanced against risks to the brevity of the assessments, which was among the strongest themes in the qualitative responses. Applying an additional layer of adaptive sampling could provide a global solution to this issue and the suggestion that substance use assessment be restricted to the past week. Respondents who regularly report high-frequency substances across weekly assessments could be issued additional weekly assessments that allow for continuous temporal coverage in lieu of the core assessments. Currently, core assessments are required to ensure that data are available to provide a continuous temporal characterization of substance use. However, further work would need to be conducted to allow for adjustments to be made because of differential measurement errors that could arise from a system where respondents are reporting at different frequencies. Development, amendments, and mobile app protocols should be made considering respondent burdens and changes to the mean square error of estimation (ie, bias and variance).

### Conclusions

Longitudinal studies of health behaviors among youth can be enhanced by using frequent self-reports administered on mobile phones. Youth will elect to participate and adhere to the assessment protocol at a high rate. However, it remains unclear as to how much variation will be observed in various subpopulations, especially in contexts where participants may not feel invested in the study. mNCANDA’s acceptability and high retention rate over a relatively long study duration for substance use and health behavior assessment app are based on brief and simple assessments, issued on a platform designed to minimize burden in responding, which incorporates reinforcement components into the app and the protocol. This app could serve an important role in filling gaps in understanding the critical developmental trajectories of substance use.

## Appendix

Multimedia Appendix 1 Mobile National Consortium on Alcohol and Neurodevelopment in Adolescence assessment items.

Multimedia Appendix 2 Qualitative instruments.

## Abbreviations

CDDR: Customary Drinking and Drug Record
GEE: generalized estimating equation
mNCANDA: mobile National Consortium on Alcohol and Neurodevelopment in Adolescence
NCANDA: National Consortium on Alcohol and Neurodevelopment in Adolescence
