# Supplementary material for: Acceptability, Validity, and Engagement With a Mobile App for Frequent, Continuous Multiyear Assessment of Youth Health Behaviors (mNCANDA): Mixed Methods Study
Source: JMIR Mhealth Uhealth. 2021 Feb 10;9(2):e24472. doi: 10.2196/24472 (PMC7904399; doi:10.2196/24472)
Supplement: Multimedia Appendix 1 [file mhealth_v9i2e24472_app1.docx]

Supplemental Table 1. mNCANDA assessment items.

| **Group Prompt** | **Item Prompt** | **Response Options** |
| --- | --- | --- |
| **Substance use (30-day)** | | |
| In the past 30 days: | How many days did you drink alcohol? | 0-30 (integer) |
|  | On days you drank, what was your AVERAGE number of standard drinks? (NA=0) | 0-20 (integer) |
|  | Most alcohol you had on an occasion? (# standard drinks)(NA = 0) | 0-20 (integer) |
|  | Days you drank VARIABLE1 or more drinks? | 0-30 (integer) |
|  | Days used marijuana/THC? | 0-30 (integer) |
|  | Days used tobacco/nicotine? | 0-30 (integer) |
| **Substance use (28-day)** | | |
| In the past 4 weeks (28 days): | How many days did you drink alcohol? | 0-28 (integer) |
|  | On days you drank, what was your AVERAGE number of standard drinks? (NA=0) | 0-20 (integer) |
|  | Most alcohol you had on an occasion? (# standard drinks)(NA = 0) | 0-20 (integer) |
|  | Days you drank VARIABLE1 or more drinks? * | 0-28 (integer) |
|  | Days used marijuana/THC? | 0-28 (integer) |
|  | Days used tobacco/nicotine? | 0-28 (integer) |
|  | What type of tobacco/nicotine product did you use? Check all that apply | cigarettes, vape, cigars, hookah, e-cigarettes, chewing tobacco/dip, snuff, snus, other, NA/NONE |
|  | On how many days did you use 'OTHERDRUGi' to get high? ** | 0-28 (integer) |
|  | Other than the above, did you use any other drugs? If so, type it here. (If you used more than one, separate each with a comma) | free-response |
|  | If you specified a new drug in the previous question, how many days did you use this new drug? (NA=0)(If you used more than one new drug, report the one you used on the most days) | 0-28 (integer) |
| **Substance use (7 day)** | | |
| During the previous 7 days: | How much alcohol did you drink? | ‡ 0-20 |
|  | What days did you use marijuana/THC? | ‡ 0-20 |
|  | What days did you use nicotine/tobacco? | ‡ 0-20 |
|  | What days did you use any other drug that was not prescribed, or not used as prescribed, by a doctor? | ‡ 0-20 |
|  | Which drug(s)? If none, leave blank. (separate each drug with commas) | free-response |
| **Access to substances** | | |
| How difficult do you think it would be for you to get each of the following types of drugs, if you wanted some? | Alcohol | Nearly Impossible, Very Difficult,  Fairly Difficult, Fairly Easy,  Very Easy |
|  | Marijuana/THC |  |
|  | Cigarettes |  |
|  | Vape juice/refills/cartridges |  |
|  | Prescription opioids (e.g., Vicodin, Codeine) |  |
|  | Amphetamines (e.g., Adderall, Ritalin) |  |
|  | Methamphetamines (e.g., meth, crystal) |  |

Supplemental Table 1. continued

| **Group Prompt** | **Item Prompt** | **Response Options** |
| --- | --- | --- |
| **Day-to-day Activity** | | |
| During the previous  7 days: | How many days did you have caffeine? | 0-7 (integer) |
|  | On the days you had caffeine, how many caffeinated beverages did you usually have per day? | 0-20 (integer) |
|  | On the days you had caffeine, how many energy drinks did you usually have (Rockstar, Monster, Red Bull, etc.) per day? | 0-20 (integer) |
|  | How many days did physical activity make you breath hard/sweat for 20+ minutes? | 0-7 (integer) |
|  | How many hours did you spend in organized sports, IN TOTAL? (sum all the hours) | 0-40, 0.5 hour increments |
|  | How many hours did you spend on schoolwork outside of class? | 0-60, 0.5 hour increments |
| On a typical day, how many hours do you: | During the previous 7 nights: How many hours did you usually sleep each night? | 0-18, 0.5 hour increments |
|  | What time did you go to bed LAST night? | 12:30 PM - 12:00 PM, 0.5 hour increments |
|  | How many minutes were you in bed before you fell asleep, last night? | 5 min, 10 min, 15 min, 20 min, 25 min, 30 min, 35 min, 40 min, 45 min, 1 hour, 1.5 hour, 2 or more hours |
|  | What time did you wake up THIS morning? | 12:30 PM - 12:00 PM, 0.5 hour increments |
|  | Rate how sleepy you felt during the day YESTERDAY (0-not at all 5-hard to stay awake) | 0-5 (integer) |
|  | Rate how sleepy you feel NOW | 0-5 (integer) |
|  | Watch TV shows or movies? | 0-24, 0.5 hour increments |
|  | Watch videos (such as YouTube)? | 0-24, 0.5 hour increments |
|  | Play video games on a computer, console, phone or other device? | 0-24, 0.5 hour increments |
|  | Video chat (Skype, Facetime, etc.)? | 0-24, 0.5 hour increments |
|  | Text on a cell phone, tablet, or computer (e.g. GChat, Whatsapp, etc.)? | 0-24, 0.5 hour increments |
|  | Visit social networking sites like Facebook, Twitter, Instagram, etc.? | 0-24, 0.5 hour increments |
| During the previous  7 days (not including today), estimate the TOTAL: | How many hours did you work at a job? | 0-60, 0.5 hour increments |
|  | How many hours did you spend in class? | 0-60, 0.5 hour increments |
|  | How many hours did you spend on schoolwork outside of class? | 0-60, 0.5 hour increments |
|  | How many hours did you spend in organized activities, hobbies, sports teams, clubs, volunteering, music lessons? | 0-60, 0.5 hour increments |
|  | How many hours did you spend in organized religious activities? | 0-60, 0.5 hour increments |
|  | How many hours did you spend in personal spiritual/religious activity (e.g., prayer, meditation, studying sacred texts, etc.)? | 0-60, 0.5 hour increments |
|  | How many times did you eat fast food in the past week? | 0-21 (integer) |

Supplemental Table 1. continued

| **Group Prompt** | **Item Prompt** | **Response Options** |
| --- | --- | --- |
| **Day-to-day Activity** | | |
| Because of your drinking or drug use during the last 7 days (NA = no): | Couldn't remember what happened, felt nauseous, threw up, or passed out... | yes/no |
|  | Didn't get homework done, didn't study for something you should have, got poorer grades on homework or a test, or missed part or all of a school day... |  |
|  | Had a problem or argument with a friend or hurt your relationship with your spouse/girlfriend or boyfriend... |  |
|  | Behaved in ways you later regretted or felt bad about... |  |
|  | Got in trouble at work or at a work related event... |  |
|  | Had a problem or argument with family members or hurt your relationship with your family... |  |
|  | Got in trouble with the police... |  |
|  | Got in trouble at school or at a school related event... |  |
| **Stress level** | | |
| During the past 7 days, how often have you: | been upset because of something that happened unexpectedly? | Never, Almost Never, Sometimes, Fairly Often, Very Often |
|  | felt that you were unable to control the important things in your life? |  |
|  | felt nervous and “stressed”? |  |
|  | felt confident about your ability to handle your personal problems? |  |
|  | felt that things were going your way? |  |
|  | found that you could not cope with all the things that you had to do? |  |
|  | been able to control irritations in your life? |  |
|  | felt that you were on top of things? |  |
|  | been angered because of things that were outside of your control? |  |
|  | felt difficulties were piling up so high that you could not overcome them? |  |
| **Complementary and alternative medicines** | | |
| During the past 7 days: | Which of these supplements did you consume: | Echinacea, Ginkgo biloba, Valerian, Kava, Passion flower, Cannabidiol (CBD), Chamomile, Melatonin, Other herbs or non-vitamin supplements, None of the above |
|  | Please list the other herb, non-vitamin supplements, or beverages that you used for health reasons or to improve your mood or functioning. | free-response |
|  | On which of the following days did you use any of the herbs or supplements that you reported on this survey? | † Every day, Sunday, Saturday, Friday, Thursday, Wednesday, Tuesday, Monday, None |
| **Quality of life** | | |
| Over the past 7 days: | How was your physical health? | Very good, Good, Neither good or bad, Bad, Very Bad, NA |
|  | How was your mental health? |  |
|  | How was your relationship with your girl/boyfriend, partner, spouse? |  |
|  | How were your relationships with your friends? |  |
|  | How did you feel about yourself? |  |

Supplemental Table 1. continued

| **Group Prompt** | **Item Prompt** | **Response Options** |
| --- | --- | --- |
| **Socialization** | | |
| On which of the past 7 days did you spend at least 1 hour: | Socializing online, where you were communicating with each other (e.g. texting, messaging, gaming with voice links) | † Every day, Sunday, Saturday, Friday, Thursday, Wednesday, Tuesday, Monday, None |
|  | Socializing in person (e.g. hanging out, recreating, dining, attending entertainment events) |  |
|  | Socializing in settings where people were using alcohol, marijuana, or using other illicit drugs? |  |
| **Physical Activity** | | |
| Over the past 7 days: | Think about the activities you do at work, as part of your house and yard work, to get from place to place, and in your spare time for recreation, exercise or sport. Vigorous activities make you breathe much harder than normal and may include heavy lifting, digging, aerobics, or fast bicycling. Think only about activities that you did for at least 10 minutes at a time. On which days did you do vigorous physical activities? | † Every day, Sunday, Saturday, Friday, Thursday, Wednesday, Tuesday, Monday, None |
|  | How much time did you usually spend doing vigorous physical activity on those days? | <10 min, 10 min, 20 min,30 min,45 min,1 hour,1.5 hour, 2 hour, 3+ hour |
|  | Moderate physical activities make you breathe somewhat harder than normal and may include carrying light loads, bicycling at a regular pace, or doubles tennis. Do not include walking. Think only about activities that you did for at least 10 minutes at a time. On which days did you do moderate physical activities? | † Every day, Sunday, Saturday, Friday, Thursday, Wednesday, Tuesday, Monday, None |
|  | How much time did you usually spend doing moderate physical activity on those days? | <10 min, 10 min, 20 min,30 min,45 min,1 hour,1.5 hour, 2 hour, 3+ hour |
|  | On which days did you do you walk for at least 10 minutes at a time? | † Every day, Sunday, Saturday, Friday, Thursday, Wednesday, Tuesday, Monday, None |
|  | How much time did you usually spend walking on those days? | <10 min, 10 min, 20 min,30 min,45 min,1 hour,1.5 hour, 2 hour, 3+ hour |
|  | How much time did you usually spend sitting or laying down (awake) per day on a weekday? | <10 min, 10 min, 20 min, 30 min,45 min,1 hour,1.5 hour, 2 hour, 3 hour, 4 hour, 5 hour, 6 hour, 7 hour, 8 hour, 9 hour, 10 hour, 11 hour, 12 hour, 13 hour, 14 hour, 15 hour, 16+ hour |

* VARIABLE1 displays a sex-defined number (Female = 4, Male=5) of standards drinks

** ‘OTHERDRUGi’ is a placeholder for the participants’ vernacular for previously reported substance use

‡ participants entered a number (0-20) for each day of the week or selected ‘None.’

† multiple response inputs accepted
