# Supplementary material for: Acceptability, Validity, and Engagement With a Mobile App for Frequent, Continuous Multiyear Assessment of Youth Health Behaviors (mNCANDA): Mixed Methods Study
Source: JMIR Mhealth Uhealth. 2021 Feb 10;9(2):e24472. doi: 10.2196/24472 (PMC7904399; doi:10.2196/24472)
Supplement: Multimedia Appendix 2 [file mhealth_v9i2e24472_app2.docx]

Supplemental Table 2. Qualitative instruments.

***Open Ended Perceptions and Feedback Items***

1.  Thinking back to the last survey that you submitted, how long did it take you to complete the survey?  ___min ___sec

2.  How many weeks ago was the last time you missed a survey: [numeric response]

*Prompt: We would like to understand your individual experiences with the mobile app.*

3.  Please, briefly describe why your last missed survey was not completed: [open ended response]

4.  What other reasons did you have for missing other mobile app surveys? [open ended response]

5.  What did you like most about using the mobile app? [open ended response]

6.  How could the questions in the mobile app be improved? [open ended response]

7.  How might we improve the frequency and timing of surveys? [open ended response]

8.  What about the mobile app did not work properly or could be improved? [open ended response]

9.  Do you have any other comments about the mobile app? [open ended response]

***Semi-structured Interview Guide***

(Aim 3 Qualitative Interview Subset)

***Introductory Prompt:*** *Thank you for participating in the initial evaluation of our new mobile app surveys. We are planning to use your feedback to guide the finalization and future development of the app. We would like to start by asking you about your experience and opinions about using your phone in the NCANDA study. This should take 10 to 15 minutes. We would like to create a transcript of your responses so we can anonymously share your views exactly as you said them. If you want us to audio record your answers, we may be able to go a little faster. We will destroy the recording after typing up your answers, and will not share the recording. It is important to let you know that there is always a risk of loss of privacy if the recordings are not kept secure. This has never happened in our lab and we will only be talking to you about the mobile app and not about any of the other things we ask about in the NCANDA study. Can we have your consent to record and temporarily store today’s conversation? [If respondent does not agree proceed and attempt to collect verbatim quotes in writing. Ask patience for time to record their responses.]*

Note to Interviewer: This is a semi-structured interview and item order can be rearranged on the fly in response to participant responses and interviewers can prioritize the importance of obtaining responses to various items based on the participants’ response and interest. Probes are suggestions. These may not be necessary to ask if a participant is providing rich responses. Other probes may be appropriate based on participants’ responses, in order to obtain an adequate coverage of the target areas of interest represented in the item.

Consent 2.  [If they agreed to recording, state:] I’m going to start recording now. I can stop at any time. The recording will only be used to make a transcription. Now that we are on tape, is that okay that I record this conversation?

1. What difficulties did you experience while using the mobile app?
2. The user interface is what is displayed on the app and its behavior when you interact with it.  What are your thoughts about the user interface of the mobile app?

    Probe 1. What features of the user-interface did you like the most?

    Probe 2. What features of the user-interface did you like the least?

1. What are your thoughts about how often assessments are issued?

1. What are your thoughts about the length of each assessment?

    Probe 1: Thinking back to the last assessment, how long did that take you to complete?

    Probe 2: About how long does it typically take to complete a survey?

1. What considerations should we make when deciding what time of day that you get notified that an assessment is due?

     Probe 3.1: In what ways were the notifications intrusive or annoying?

1. How do you feel about us sending reminder notifications and texts if you don't initially respond?

     Probe 1: What should we consider when deciding how long to wait until sending the first and second reminders?

1. (Only ask if surveys were missed) Please, briefly describe why your last missed survey was not completed:

Subquestion 1: When was that survey missed?
Probe 1: Your last missed assessment was on DATE/DAY/Days Ago, do you recall getting assessment notifications on that day?

Subquestion 2: What other reasons did you have for missing surveys?

Subquestion 3: How could we change things to have you respond to most of the surveys?

1. In what ways does using the mobile app change the way you behave?

Probe 1: In what ways does it change your substance use?

Probe 2: In what ways does it change your daily activity?

1. How did you feel about the way that credits for participation were issued?

Probe 1: How did you feel about bonus/ramped up credits for better response rates?

56.  If we were to continue using the mobile app in the study over the next several years, what do you think it would take for you to participate and respond to most of the assessments?

Probe 7.1: What could we change about the frequency of assessments?

Probe 7.2: What could we change about the length of the assessments?

Probe 7.3: What could we change in the way we pay for your responses?

57.   What are your feelings about the questions that were asked on mobile app?

Probe 3.1: Were there any questions on the app that you think might make people uncomfortable to have asked on their mobile phone?

Probe 3.2: Were there questions that were hard to understand or didn’t make sense?

Probe 3.3: Did you wonder about the value of any of the questions that you were asked?

Probe 3.4: Were there questions you think we should be asking or asking in a different way?

58.  Do you have any other comments that you would like us to know?
